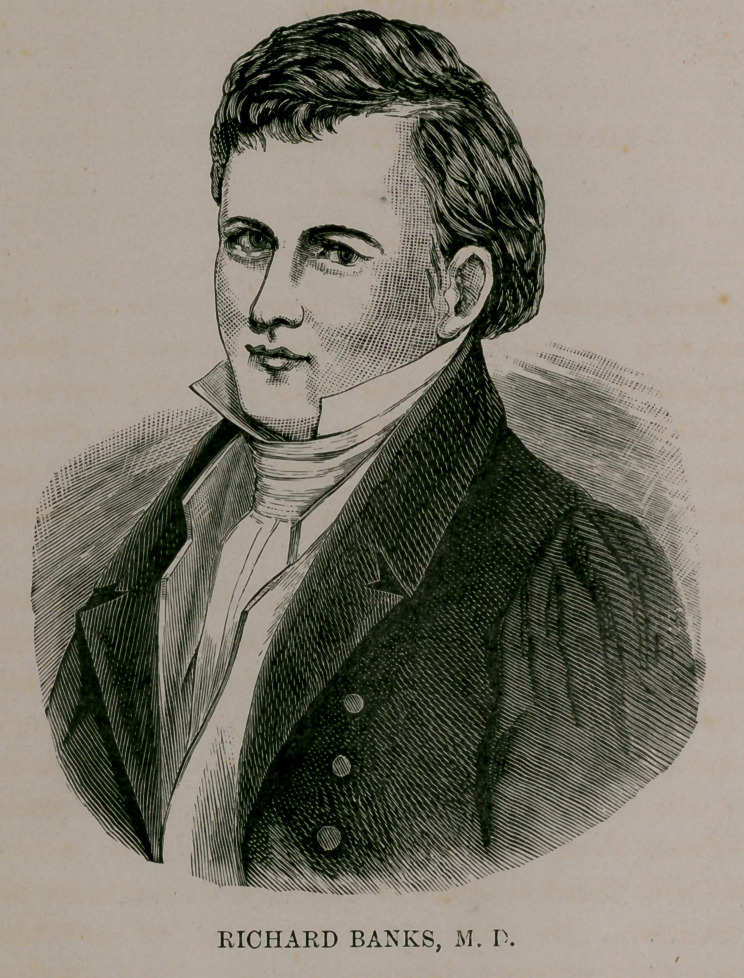# Our Portrait Gallery

**Published:** 1885-07

**Authors:** 


### OUR PORTRAIT GALLERY.

### RICHARD BANKS, M. D.

The eastern portion of the State of Georgia bordering on the Savannah river was quickly settled up after the close of the Revolutionary war by hardy, industrious, enterprising emigrants from Virginia and North Carolina. They left the impress of their character and civilization upon the entire commonwealth, and from their descendants have come many of the most distinguished citizens of the Empire State of the South.

Amongst the new comers was Ralph Banks, who emigrated from North Carolina and settled in Elbert county. Besides the usual motives for removal, the hope of bettering his fortunes, and laying for his children the foundation of future prosperity, Ralph Banks had another object. His venerable father had become infatuated with the charms of a youthful maiden of the Old North State—Psyche Pretty by name. The son, believing that absence conquers love, placed hundreds of miles of unbroken forest between the lovers. His schemes were unavailing; the aged Adonis, after a short sojourn in Georgia, returned to North Carolina and married the Pretty Psyche and founded another family of Banks, who still flourish in the far West.

Ralph Banks was an intelligent, successful and prosperous Georgia farmer, cultivating tobacco and rolling the hogsheads, in which it was compressed, to market in Augusta, until the invention of the cotton gin furnished him a more profitable staple.

His high moral character and genial manners attracted to his hospitable home many visitors. His house became the resting-place of the early Methodist ministers. Bishop Asbury in his journal mentions that, on one of his visits to Elbert county, he stopped with Ralph Banks, whose handsome and healthy wife—thirty-six years old—had twelve children. From this family sprang some of the leading Methodist families in Georgia. Of eight sons, every one of them arrived at distinction; several of them acquired great wealth, and all of them preserved their Methodist connections. Their descendants to-day are numerous and influential people in the State.

One of these eight brothers—Richard Banks, the subject of this sketch—was born at the paternal homestead, in Elbert county, in 1784. After such preliminary training as the schools in the vicinity afforded, he was transferred to Athens, the seat of the State University, and pursued his classical studies in the class with which graduated the Hon. Joseph Henry Lumpkin, the great Chief Justice of the Supreme Court of the State of Georgia.

Having selected medicine as a profession, he studied it diligently and successfully under private instruction. He then matriculated in the University of Pennsylvania, and after a pupilage of two years in it he graduated M. D. in 1820.

After another year's residence in the hospital, he returned to Georgia and established himself in practice in the village of Ruckersville, in his native county.

With his fine opportunities, his thorough preparation, his sturdy intellect, his talent for original observation, his cool courage in adopting and executing the conclusions of his judgment, mingled astonishment and regret are excited at his not having chosen a wider theatre for the growth and display of his extraordinary powers. His innate modesty, his scorn of all the little arts of the charlatan, sometimes employed to attract notice, his aversion to every appearance of a desire to court notoriety, doubtless influenced his determination. In the obscure village, remote from any large town, as an humble country doctor, he achieved an enviable distinction, worthy of lasting commemoration.

As a practitioner of medicine, his fame spread rapidly and widely. All over the upper part of Georgia and South Carolina his counsel was sought by physicians and by the laity, and the country is still filled with the traditions of his skill and beneficence. Unhappily, nothing but tradition remains of his intuitive perceptions of the exact essence of disease, and of his wondrous power to stay its progress. Medical journals in the South were then unknown, and the busy doctor had little time and less inclination to make a permanent record of his experience.

As an operative surgeon he early gained in the South the highest rank, and stood for many years confessedly without an equal. Fresh from the lessons of the adroit surgeons who then controlled the hospitals in Philadelphia, reliant on his own common sense and personal tact, he felt competent to perform any needed operation, and taking the whole of surgery for his province, he shrank from not even the most difficult.

Dr. Banks preserved no notes of his cases, and never, it is believed, published an account of any of them. They were in vast numbers, and of every possible variety. No one within a hundred miles of his residence thought of applying to any one else, if his assistance was possible. The loss of so vast a volume of experience is a public calamity. A single case, related by a non-professional eye-witness, follows in his own language:

"It was a child three or four years old; the upper lip and roof of the mouth were cleft open, so far back that there was no bridge to the nose, and you could see far down the throat. It was really hideous to look at. In those days anesthetics were not used. Dr. Spalding and some one else held it still during the operation. I would remark that I was as much impressed at the time by the heroism of the child's mother (she held it in her lap all the time) as I was by the Doctor's coolness and steadiness of hand. I was much affected, and of course cannot undertake to speak positively of the *modus operandi.* I recollect, however, that the inner edges of the opening were scarified; the bones of the upper jaw were divided with an instrument something like a pair of scissors; the parts were forced together with strong ligatures. The point of the nose projected considerably; this was scarified and turned down to make a bridge for the nose, and fastened in place either by adhesive strips or thread; can't remember how long the operation lasted; very short, I think.

"I saw the child some weeks after when it was brought for the Doctor to see it. The mouth was almost natural; looked like it had been marked by a fall, but had cured up. The bridge of the nose had not adhered well at the bottom; this was remedied by scarifying, and forced to adhere by being held with stitches. I remember well that it was quite a good looking child when I last saw it, and no one would have supposed it was the hideous thing brought to be operated on.

"Dr. Spalding wrote a report of the case for one of the medical journals, and, on submitting it to Dr. Banks, he would not consent to its publication. You know how modest he was. He laughed, and said he could not bear to see himself in print, especially in the florid style Dr. Spalding had employed."

Of the great number of such cases as usually occur in the practice of surgeons of wide-spread fame, but little notice was taken. Dr. Banks had a horror of notoriety, and seldom spoke, even privately to his friends, of the extent or success of his business. It is known, however, that every surgical disease brought to his notice elicited his prompt attention, and when the implements in use, or accessible, were not adequate to the emergency, he possessed inventive skill enough to devise and have made others suited to his purpose.

One of his earlier triumphs was the successful removal of the parotid gland, at a period when the best anatomists and surgeons of this and other countries were hotly discussing the question of its possibility. The details of this operation are all lost to the profession, except the fact that he dislocated the inferior maxillary articulation, in order to facilitate it.

The operations which gave him greatest celebrity, from their frequency and success, were those for cataract and for stone in the bladder. For many years he was the only surgeon in a large extent of country who attempted either, and patients sought him from great distances. How many cases of cataract he operated upon is not certainly known, nor is the exact percentage of recoveries—the number of both reported seems large, and cannot now be verified—but it is quite certain that he was generally successful.

Sometime before his death, he stated to a friend that he had performed lithotomy sixty-four times, with but two unsuccessful cases. Whether this long list was subsequently added to is unknown.

Dr. Banks' manner of making both of these capital operations was different from the methods now generally practiced. He never removed a cataract by extraction, but always by couching or absorption. He thought these methods gave better results, and were safer, inasmuch as, in case of failure of the first attempt, it could be repeated as often as might be necessary, whereas the failure of an operation by extraction necessarily resulted in permanent loss of vision. These reasons had greater weight before the discovery of anesthetics than now. The use of them has given greater facility to the operation by extraction, and it is now generally performed by the most distinguished oculists.

Lithotomy he always performed with the gorget, an instrument almost unknown to the present generation of surgeons. The lithotome, double or single, or the bistoury, in adroit hands, have supplanted it, and are now universally employed; but by no new instrument or other method have better results been secured than by the gorget, in the hands of Dr. Banks, or by Professor Dudley, of Kentucky, who followed the same method. In this, as in many other instances, that instrument is best which the operator can most skillfully handle. Statistics do not declare decisively in favor of either.

In reviewing Dr. Banks' professional career, with the unfavorable surroundings as a country doctor, we are filled with admiration at his magnificent success, and share the regret of a personal friend, that his characteristic modesty prevented his removal to some large city, where his skill and learning would have made for him a world-wide reputation ; but, like his near neighbor, Dr. Crawford Long (another Georgia country doctor), he did not seem to comprehend the importance of what he did for mankind.

In 1832, Dr. Banks removed to the village of Gainesville, in Hall county, where many of his professional triumphs were achieved, and where he resided until his death in 1856.

Gainesville was within a few miles of the territory then occupied by the Cherokee Indians. The small-pox prevailed amongst them at one time. Dr. Banks was employed by the Federal Government to visit and carry to them the knowledge and benefits of vaccination; he performed this duty faithfully, and gave them also the benefits of his surgical skill. He greatly enjoyed the wonder of these simple people at the restoration to sight of many of them who had been blind for years. "The Great Medicine

Man" they thought possessed of superhuman power and superhuman beneficence.

At the age of thirty-six—in 1830—Dr. Banks married the widow of Mr. George A. Dawson, (a nephew of Senator Dawson). As Miss Martha Butt, she was one of the most admired belles of the county of Warren. She was only in her twenty-first year at the date of her second marriage. Twenty-five years of married life, and a like period of widowhood, she passed in Gainesville. She was perhaps the best known and most beloved and esteemed lady in Upper Georgia. Her remains are fitly buried by the side of her husband, in the Methodist church-yard, the church of their choice and of their affections.

Four children, the offspring of this marriage, survive to revere the memory and imitate the virtues of their noble ancestry.

Dr. Banks acquired and enjoyed an ample fortune. His prudence, judgment and good sense were as remarkable in the conduct of his pecuniary, as of his professional business, and enabled him to leave to his widow and to his children a good estate.

In honor of his memory, the General Assembly of the State of Georgia gave to a subdivision of her territory the name of "the County of Banks."

As an incident of general interest, it may be added, the first and most beautiful of the diamonds, the product of Georgia, were found upon tbe mining property long owned and worked by Dr. Banks. A description of one of them is copied from a Gainesville newspaper :

"The second diamond found in this mine was found by Mr. Thomas Bell. It was a beautiful gem, in its natural state, so much so that it was contemplated to set it without cutting. It was sent to London twice and to Paris once to ascertain its value. It was pronounced a diamond of the first water, and was valued at three hundred and fifty dollars.

"This diamond has been seen by many of our citizens. It is worn by one of the fairest of the daughters of Gainesville, Miss Sue Banks, who has herself been appropriately called the 'Diamond of Hall County.' This lady, the youngest daughter of Dr. Banks, now Mrs. Sue Pledger, a citizen of Atlanta, still wears the gem, and retains enough of its sparkling qualities to show the appropriateness of the sobriquet, though she has generously shared with her beautiful daughters much of its brilliancy."

NOTE.—The picture from which our engraving is made is the only one in existence of Dr. Banks, and was taken at an early period of his career.

## Figures and Tables

**Figure f1:**